# Behavioural plasticity in the early breeding season of pelagic seabirds - a case study of thin-billed prions from two oceans

**DOI:** 10.1186/s40462-019-0147-7

**Published:** 2019-01-22

**Authors:** Petra Quillfeldt, Henri Weimerskirch, Juan F. Masello, Karine Delord, Rona A. R. McGill, Robert W. Furness, Yves Cherel

**Affiliations:** 1 Department of Animal Ecology and Systematics, Justus Liebig University Giessen, Heinrich-Buff-Ring 26, 35392 Giessen, Germany; 2 UMR 7372 du CNRS et de l’Université de La Rochelle, Centre d’Etudes Biologiques de Chizé, 79360 Villiers-en-Bois, France; 3 NERC Life Sciences Mass Spectrometry Facility, Scottish Universities Environmental Research Centre, East Kilbride, Glasgow, G75 0QF UK; 4 College of Medical, Veterinary and Life Sciences, University of Glasgow, Glasgow, G12 8QQ UK

**Keywords:** *Pachyptila belcheri*, Breeding schedule, Central-place forager, Foraging ecology, Geolocation, Stable isotopes, Tracking

## Abstract

**Background:**

In long-lived seabirds that migrate large distances independently of each other, the early part of the breeding season is crucially important for a successful reproductive attempt. During this phase, pair bonds are re-established and partners coordinate their breeding duties. We studied the early breeding season in Thin-billed prions *Pachyptila belcheri* breeding in the Atlantic Ocean (Falkland/Malvinas Islands) and Indian Ocean (Kerguelen). Despite overlap in the wintering areas, these two populations exhibit differences in their timing and direction of migration. We hypothesised that these differences would influence behaviour during the early breeding season.

**Results:**

In line with our hypothesis, we found very strong differences in colony attendance patterns. Thin-billed prions of the Falkland population spent the late winter period over shelf waters close to the colony, first arrived back at the colony in September, and attended the nests interruptedly for one month, before departing on a pre-laying exodus. In contrast, Kerguelen birds remained in the non-breeding areas until mid-October and spent much less time attending the burrow before their pre-laying exodus. Despite this asynchronous arrival to the two colonies, the subsequent patterns resulted in remarkably synchronous incubation in both populations, with males taking on the first long incubation shift in late November, whereas females returned to sea soon after egg laying. During the pre-laying exodus and incubation, Thin-billed prions from the Falklands spread north over the Patagonian Shelf, while prions from Kerguelen travelled much further, reaching southern oceanic waters and moved at faster speeds (> 400 km per day). Although prions from Kerguelen moved much further, their isotopic niches were considerably narrower, suggesting a stronger dependence on Antarctic waters.

**Conclusions:**

The study thus suggests that Thin-billed prions show a high intraspecific plasticity in their use of either neritic or oceanic waters during the early breeding season. Breeding birds from the Falkland Islands can exploit an extensive shelf area, while Kerguelen birds have adapted to the need to forage in distant southern open waters. This difference in foraging ecology may thus have shaped the phenology of the early breeding phase.

**Electronic supplementary material:**

The online version of this article (10.1186/s40462-019-0147-7) contains supplementary material, which is available to authorized users.

## Background

Migratory animals can benefit from productive feeding sites during the non-breeding season. However, they should also time their return to the breeding sites well, in order to maximize their fitness [1]. They should arrive at the breeding grounds in time to set up a territory or defend a breeding site, reunite with or attract a mate and to coincide with high food availability during the reproductive period [2].

Despite stable pair-bonds during the breeding season, many migratory birds spend the non-breeding season independently of their breeding partner (e.g. [1–4]). During the early part of the breeding season, pair bonds are re-established and partners coordinate their breeding duties. This stage is therefore crucially important for the maintenance of pair-bonds [1] and thus, a successful reproductive attempt. This is especially true in pelagic seabirds such as Procellariiformes, because despite their long stable pair-bonds and high reproductive investment into their single offspring per year, they migrate over thousands of kilometres independently of each other (e.g. [2]).

The transition from the non-breeding to the breeding season starts with the re-occupation of the nest site, pair-bond re-establishment and mating. During this phase, Procellariiformes typically spend a high proportion of their time ashore, where they fast and lose weight [5]. To recover body condition and in the case of the females, to feed up for egg production, both male and female petrels often fly long distances to areas of high resource abundance during an extended foraging trip before laying - the pre-laying exodus or ‘honeymoon period’ [6]. As soon as the female returns from the pre-laying exodus, she lays a single egg, and departs again, while the male usually takes the first long incubation shift [5].

A fine-tuned timing of arrival back to the colony, e.g. synchronous within colonies and co-ordinated with the breeding partner, will ensure that birds can successfully breed after re-occupation of their breeding nest or burrow and reunion with their long-term partner. The pre-breeding time spent with the breeding partner may be important for strengthening the re-established pair bonds and ensuring successful copulation [7]. For example, Balearic shearwaters *Puffinus mauretanicus* visited the colony on consecutive nights in the pre-breeding season, until they coincide with their partner and often spent the following day in the cave together [8]. Wandering Albatrosses *Diomedea exulans* with better body condition arrived earlier and spent more pre-breeding time at the colony and successful birds spent more time with their breeding partners [9].

The aim of the present study was to investigate plasticity in the behaviour during the early part of the breeding season in a Southern Ocean petrel, the Thin-billed prion *Pachyptila belcheri*, from the arrival at the colony to the first incubation shift. Procellariiformes (petrels, shearwaters, albatrosses) are the most pelagic of the seabirds, and like most Procellariiform species (e.g. [10, 11]), Thin-billed prions migrate hundreds or thousands of kilometers away from the breeding colony for part of the year [12, 13]. Thin-billed prions have two main breeding sites more than 8000 km apart, on the Falkland/Malvinas Islands in the south-western Atlantic and in the Kerguelen Archipelago in the Indian Ocean. Using geolocation loggers, we found that these two populations exhibit differences in their timing and direction of migration [13]: Falkland birds migrated more than 3000 km eastwards, while Indian Ocean birds migrated westwards, resulting in an overlapping nonbreeding area for the two populations in the eastern Atlantic sector of the Southern Ocean. Falkland birds returned to the Patagonian Shelf after 2–3 months, while Kerguelen birds remained in the nonbreeding area for seven months, before returning to their nesting grounds highly synchronously and at high speed [13].

We hypothesised that these differences, and the different environmental conditions experienced, would influence their behaviour during the early breeding season. In the present study, we specifically aimed to: (i) compare colony attendance patterns and trip durations of birds breeding in the Falklands and Kerguelen, (ii) determine the foraging areas used by the two populations during the pre-laying exodus (i.e. the time spent away from the colony before egg-laying) and early incubation using bio-logging, and (iii) examine the trophic ecology during the early breeding season using stable isotope data from tracked birds.

## Methods

### Study species and sites

Thin-billed prions breed on islands off South America and in the Indian Ocean; there are several million birds in the Falkland and Kerguelen islands, a smaller population on Isla Noir (southern Chile) and a very small number (10–20 pairs) on the Crozet Islands [14]. They show the typical procellariiform pattern of a single-egg clutch and slow chick development. Thin-billed prions feed mainly on crustaceans during the breeding season and show some flexibility in diet within and between years [15–17].

To investigate spatial movements, we attached leg-mounted miniaturized saltwater immersion geolocators (MK10, developed by British Antarctic Survey, Cambridge, UK) to breeding adult Thin-billed prions over three years at New Island, Falkland/Malvinas Islands (51°43′S, 61°18′W) and during one year at Île Mayes, Kerguelen (49°28’S, 69°57′E, for sample sizes, see Table 1).

**Table 1:** Geolocator deployment and recovery times and sample sizes for Thin-billed prions *Pachyptila belcheri* from the Falkland Islands (FLK) in 3 years and Kerguelen (KER) in 2012

Year (Island)	Deployment (N, dates)		Recovery(N, dates)		Year-round tracks
2010 (FLK)	25	27/11/09–11/2/2010	20	17/12–29/12/2010	20
2011 (FLK)	20	25/12–31/12/2010	14	04/12–11/12/2011	9
2012 (KER)	29	13/01–18/01/2012	19	26/11–03/12/2012	15
2013 (FLK)	20	10/12–19/12/2012	11	29/11–14/12/2013	6

Nests were selected according to accessibility, and at New Island, the presence of individuals known from previous years, to maximize the chances of recapture. Newly marked nests were used at Île Mayes because the known nests are part of a standard monitoring programme. The birds were captured by hand at marked nests during incubation. The geolocators weighed 1 g and were fixed to plastic leg bands. The total weight including ring, self-amalgamating tape and a cable tie was 1.5 g (< 1.5% of the mean body mass - 130 g - of Thin-billed prions). A detailed study found no evidence for any substantial impact of the geolocators on Thin-billed prions: breeding performance was unaffected in the season of attachment or following recovery; eco-physiological measurements suggested that adults adapted to the higher load; and the similarity in stable isotope ratios in blood and feathers of instrumented adults and controls indicated that general diet and distribution was unaffected [18].

Tagged individuals were marked with numbered steel rings on the other leg. A blood sample for sex determination was taken from the wing vein and stored on FTA cards. Burrows were revisited and devices retrieved during incubation in the following season (Table 1).

Because several loggers stopped recording several months before device recovery, the final sample sizes for year-round tracks, which were used in this study, were smaller than for recovered data sets (Table 1). The GLS recorded data from a single pre-breeding period for each individual. Several pairs were included (Falklands 2010–11: 8 pairs, 2013: 1 pair, Kerguelen: 2 pairs), but as their movement patterns were independent (Additional file 1: Figure S1), they were included as independent data., The number of pairs with successful data retrieval was not great enough, especially for Kerguelen birds, to analyse data at the pair level.

### Data processing

Geolocators provide two positions per day based on light levels, with an accuracy of approximately 186 ± 114 km [19]. Light data were analysed using the BASTrak software suite (British Antarctic Survey, Cambridge, UK). TransEdit was used to check for integrity of light curves and to determine dawn and dusk times, and Locator to estimate the latitude from day length and longitude from the time of local mid-day relative to Greenwich Mean Time. We assumed a sun elevation angle of − 3.5°, based on known positions obtained during pre- and post-deployment calibration of the loggers at the colony. All estimated locations were examined visually in a geographical information system (GIS) and any unrealistic positions in the year-round tracks – either associated with interference to light curves at dawn or dusk, or in proximity to equinoxes when latitudes are unreliable - were excluded from further analyses. However, this did not affect the honeymoon or incubation periods, when data were complete.

Trips to sea were distinguished from periods in the burrow by examining the light and immersion data. Following [8], the occurrence of complete daytime darkness in the logger trace allowed identification of days spent in the colony, whilst sustained periods of night-time dryness in the immersion data (> 2 h) allowed identification of visits to the burrows during the night. The pre-laying exodus was an obvious phase lasting 14–41 days when the bird was at sea, which preceded the first incubation shift. It was also possible to determine the day of first arrival in the colony, the period from first arrival to the start of the pre-laying exodus (hereafter, the “pre-exodus phase”), and the total number and proportion of days spent in the burrow during this phase. In addition to determining only days at the colony and days at sea (e.g. [13]), we distinguished another category, namely days at sea followed by nights in the burrow. Days away from the nest during the incubation shifts indicated egg neglect (also termed intermittent incubation), a common strategy in petrels and some other offshore feeders (e.g. [20]).

Changes in distribution between phases of the breeding season were examined using kernel analysis of filtered locations [19]. The non-parametric fixed kernel density estimator was used to determine density contours. Kernel densities do not require serial independence of observations when estimating foraging ranges [21]. Kernel analyses were performed in a Lambert equal-area azimuthal projection centred on the South Pole using ArcGIS 9.3 (ESRI, Redlands, CA, USA) and Hawth’s Analysis Tools [22]). As Thin-billed prions cover large distances compared to the error of the GLS devices, the total distance travelled during foraging trips was calculated in the same projection.

### Sex determination

The sex of each bird in this study was determined through PCR using primers 2550 and 2718 that amplify sections of the sex-linked chromo-helicase-DNA binding (CHD) gene [23]. DNA was extracted from 50 μl blood using a Qiagen DNAEasy blood purification kit (Qiagen, Hilden, Germany). Each reaction was carried out in 25 μl, containing 10 ng template DNA, 1 × PCR buffer, 0.1 mM DNTPs, 2.5 mM MgCl2, 0.2 μM of each primer and 0.1 U Taq polymerase (Firepol, Soilis Biodyne, Tartu). Thermocycling consisted of an initial denaturation step of 2 min at 94 °C, followed by 35 cycles denaturation at 94 °C for 30 s, annealing at 54 °C for 30 s, extension at 72 °C for 1 min, and ended with two expansion steps of 42 °C for 1 min and 72 °C for 10 min. PCR products were visualised on a 2% agarose gel, with a single band at ~ 650 bp indicating a male, and two bands at ~ 450 and ~ 650 bp indicating a female.

### Stable isotope analyses

Carbon and nitrogen isotopic studies from the Southern Ocean clearly show δ^13^C values of seabirds correspond to the location of their foraging habitats [24–26] and their δ^15^N values increase with trophic level [27]. Stable isotope values of blood (full blood in Kerguelen birds, red blood cells in Falkland birds) and feathers grown during the non-breeding period (lower back/rump feathers in Kerguelen birds, a small segment of the inner vane of the innermost primary in Falkland birds) were collected during the retrieval of the geolocators.

Whole blood δ^13^C and δ^15^N values are very close to those of blood cells because blood cells contain more organic matter than plasma [28]. Bird whole blood/blood cells have a turnover time of ca. 4 weeks [29]. Thus, samples taken after recapture of the birds carrying GLS were representative of the early breeding season, in particular the pre-laying exodus. Feathers represent the time of the moult, which occurs in the winter quarters in polar waters after the breeding season [30]. While primaries are replaced during the core moulting period as determined by decreased flight activity [30], body feathers of Procellariiformes are replaced gradually over several months of the non-breeding period. However, Thin-billed prions from Kerguelen remain in the same area throughout the non-breeding period, and thus, their body and primary feathers should be comparable. Blood and feathers have different isotopic discrimination factors that must be taken into account when comparing their δ^13^C and δ^15^N values [31]. Hence, Antarctic δ^13^C values were those < 22.5‰ and < 21.2‰ for blood and feathers, respectively [25].

Tissue sub-samples were weighed (0.4–0.7 mg) with a microbalance, packed in tin capsules. Carbon and nitrogen isotope ratios were measured simultaneously by continuous-flow isotope ratio mass spectrometry, as described earlier [13] at the LIENs laboratory at the University of La Rochelle, France and the NERC Life Sciences Mass Spectrometry Facility, Glasgow. Replicate measurements of internal laboratory standards indicated measurement errors < 0.20 ‰ for δ^13^C and δ^15^N. All stable isotope ratios are expressed in δ notation as parts per thousand (‰) deviation from the international standards Vienna-Pee Dee Belemnite (carbon) and AIR (nitrogen).

### Statistical data analyses

Statistical analyses were conducted using SPSS 11.0. We tested for normality using Kolmogorov-Smirnov tests and by checking plots of the data. General linear models (GLMs) based on Type III sum of squares were used to test for differences in the timing and distribution of each parameters among years and sexes. We carried out a separate GLM for each timing and distribution parameter, with sex and year included as categorical independent variables (“factor”). Initially, we included the interaction between the two factors, but this was removed if non-significant (e.g. [32]). As a measure of effect sizes we used partial Eta-Square values (*η*^2^; i.e. the proportion of the effect + error variance that is attributable to the effect) in case of variables and covariates tested with a GLM. The sums of the partial Eta-Square values are non-additive (e.g. https://www.uccs.edu/lbecker/glm_effectsize). Throughout this study all means are given ± S.D.

Isotopic niches were compared using the SIAR package in R and the metrics suggested by [33] and the Bayesian approach based on (small sample size corrected) standard ellipse metrics [34].

Three different years were included for the Falkland Islands, but as both a previous study [35] and the present data indicate a consistent behaviour across years, the data were pooled for comparisons between the populations.

## Results

### Return to breeding colony

**Fig. 1:**
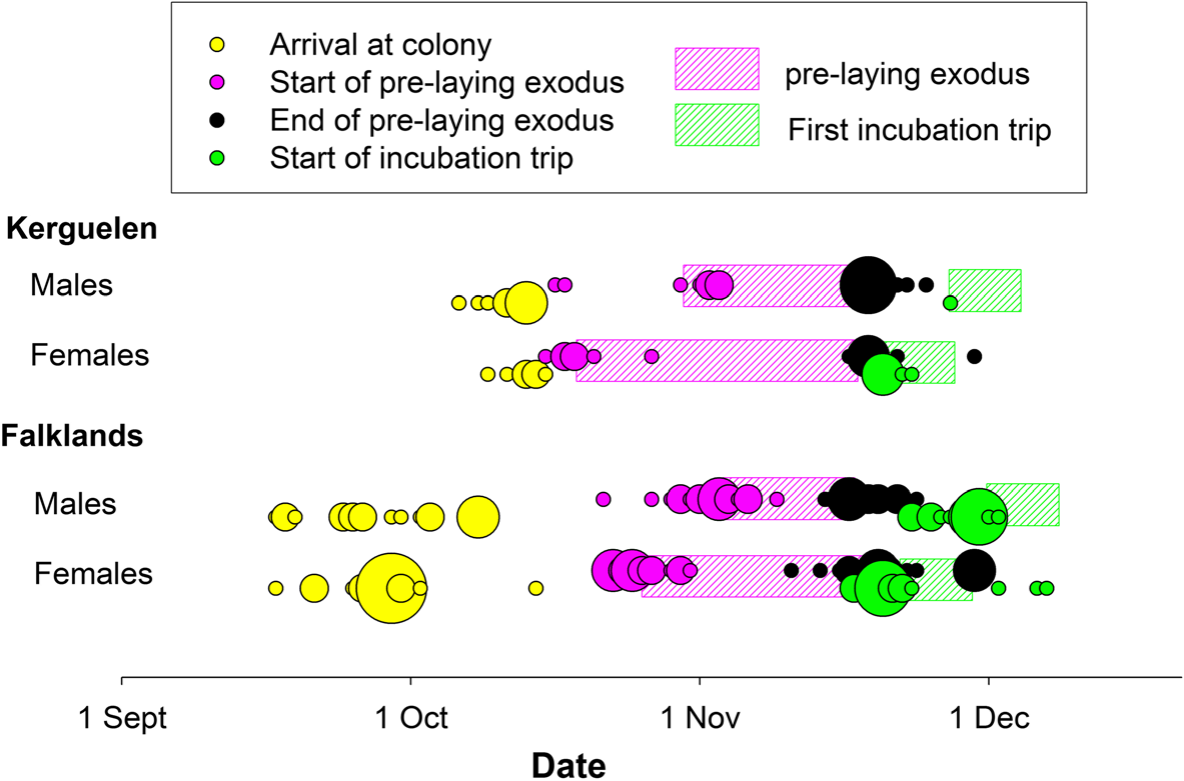
Early breeding season chronology of Thin-billed prions *Pachyptila belcheri* from the Falkland Islands (*N* = 17 males, 18 females) and Kerguelen (*N* = 8 males, 7 females). Larger dots mark more individuals starting or ending a phase on the same day

**Fig. 2:**
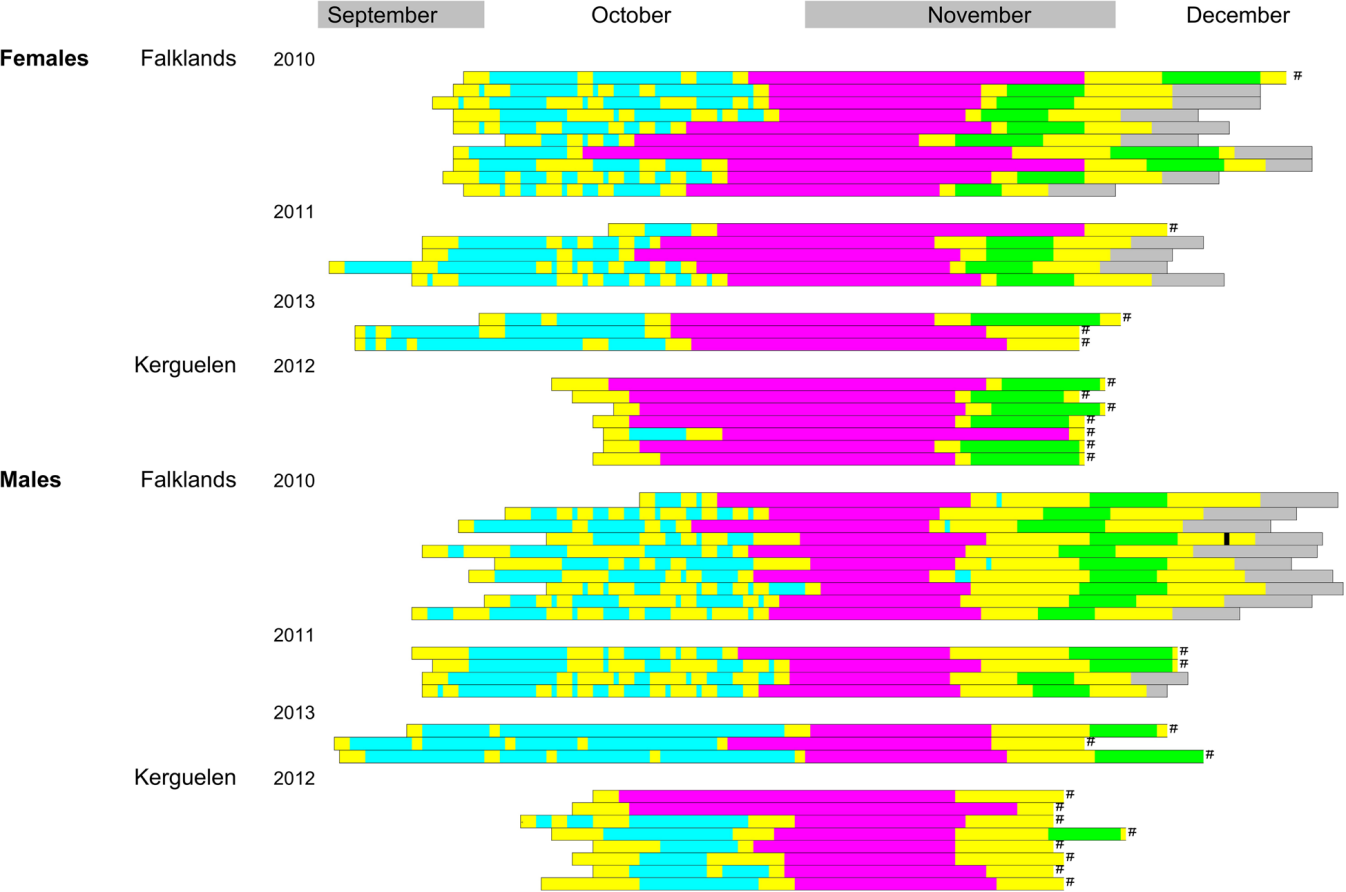
Individual early breeding season chronologies of Thin-billed prions from the Falkland Islands (*N* = 35) and Kerguelen (*N* = 15). Color bars show the timing and duration of the subsequent phases from arrival to the colony (first yellow block) until the end of the second foraging trip in incubation (marked in grey) or earlier recapture (marked with #). Colony attendance before the pre-laying exodus consisted of shifts of 1–9 days in the burrow (in yellow) alternating with foraging trips of 1–13 days (in turquoise), followed by the pre-laying exodus (in pink). After return from the pre-laying exodus, the birds spent 1–16 days in the burrow (in yellow), followed by a first foraging trips (marked in green). In 2010, 1 of 10 of the males at New Island showed some egg neglect (marked black), lasting for a single day (the birds left one night and returned the subsequent night)

Thin-billed prions arrived back to the Falklands from 16 September to 16 October (mean: 28 September ±7 days) and to Kerguelen 2 weeks later (Fig. 1, Table 2), from 5 to 14 October (mean: 11 October ±3 days). The arrival date did not differ between sexes (Fig. 2, Table 2).

**Table 2:** Effect of colony and sex on timing of arrival and pre-laying attendance patterns during the pre-exodus phase of Thin-billed prions *Pachyptila belcheri* from the Falkland Islands and Kerguelen

Dependent	Independents	F	P	Effect size
Arrival date	**Colony**	**47.93**	**< 0.001**	**0.505**
	Sex	0.03	0.856	0.001
Total duration of pre-exodus phase (days)	**Colony**	**40.12**	**< 0.001**	**0.461**
	**Sex**	**11.03**	**0.002**	**0.190**
Cumulative period spent in the burrow (days)	**Colony**	**13.23**	**< 0.001**	**0.220**
	**Sex**	**7.83**	**0.007**	**0.143**

Significant *p*-values (*P* > 0.05) are marked bold. As a measure of effect sizes, we report partial Eta-Square values (η^2^). None of the interactions were significant (*N* = 50 birds)

**Fig. 3:**
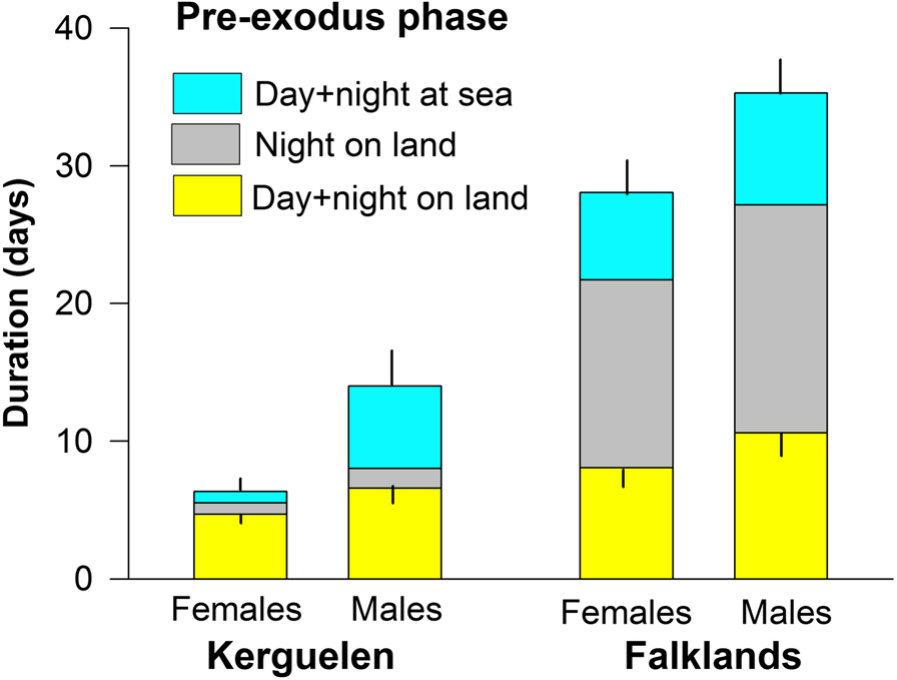
Duration of the pre-exodus phase (separate for time on land and intervals at sea) in male and female Thin-billed prions *Pachyptila belcheri* from the Falkland and Kerguelen Islands (means, SD)

The pre-exodus phase (i.e. from first arrival at the colony to departure for the pre-laying exodus) lasted 3–45 days, with significant differences between the colonies and sexes (Table 2, Fig. 3). The pre-exodus phase consisted of shifts of 1–9 days in the burrow (Fig. 2, in yellow), alternating with foraging trips lasting 1–13 days (Fig. 2, in turquoise). Especially in the Falklands, Thin-billed prions also interspersed a considerable number of days at sea with nights in the burrow.

The colony attendance between the first arrival to the colony and the pre-laying exodus was longer in males compared to females, and in birds from the Falklands compared with Kerguelen (Table 2, Fig. 3). The pre-exodus phase lasted 34.3 ± 6.7 days and 13.0 ± 6.9 days in males from the Falklands and Kerguelen, respectively, and 27.1 ± 6.2 days and 5.3 ± 3.2 days in females from the Falklands and Kerguelen, respectively.

During this time, males spent a cumulative period of 15.5 ± 4.3 days and 6.5 ± 3.2 days on land in the Falklands and Kerguelen, respectively, equivalent to 48 and 61% of the total pre-exodus phase, whereas females spent a cumulative time of 12.0 ± 3.2 days and 4.1 ± 1.4 days on land in the Falklands and Kerguelen, respectively, equivalent to 45 and 87% of the total pre-exodus phase. Six of seven females from Kerguelen spent all of their pre-exodus phase on land, without a foraging trip (Fig. 2), and left directly for their exodus. Overall, Kerguelen birds spent less time on land in absolute terms, but more time in relative terms.

### Pre-laying exodus

Thin-billed prions had a synchronous timing of departure for their pre-laying exodus at both sites within and among colonies (Fig. 1, Table 3). Females departed first, on 20 October (± 5 days) while males departed on average on 28 October (± 5 days) in both colonies. The departure date differed between sexes, but not between colonies (Table 3).

**Table 3:** Effects of colony and sex on timing and duration of the pre-laying exodus in Thin-billed prions *Pachyptila belcheri* from the Falkland/Malvinas Islands and Kerguelen

Dependent	Independents	F	*P*	Effect size
Departure date	Colony	2.69	0.107	0.054
	**Sex**	**28.72**	**< 0.001**	**0.379**
Trip duration (days)	**Colony**	**4.7**	**0.035**	**0.091**
	**Sex**	**34.4**	**< 0.001**	**0.422**
Return date	Colony	0.78	0.381	0.016
	Sex	1.53	0.223	0.031
Cumulative travel distance (km)	**Colony**	**30.40**	**< 0.001**	**0.393**
	**Sex**	**12.51**	**< 0.001**	**0.210**
Maximum distance from colony (km)	**Colony**	**19.30**	**< 0.001**	**0.271**
	Sex	0.18	0.671	0.003
Travel speed (km/day)	**Colony**	**21.95**	**< 0.001**	**0.318**
	Sex	0.17	0.682	0.004

Significant *p*-values (*P* > 0.05) are marked bold. As a measure of effect sizes we report partial Eta-Square values (η^2^). None of the interactions were significant (*N* = 50 birds)

Females engaged in pre-laying exoduses of 27.7 ± 6.1 days in the Falklands and longer trips of 31.6 ± 2.8 days in Kerguelen. Males carried out significantly shorter trips, of 19.2 ± 3.3 days in the Falklands and 22.4 ± 8.4 days in Kerguelen (Fig. 1, Table 3). The longer trip duration in females compensated for the earlier departure, such that females and males returned to the colonies on similar dates, on 18 November (± 4 days) on average, in both colonies (Table 3, Fig. 1).

During the pre-laying exodus, males travelled 5552 ± 1981 km from the Falklands and much further, summing 9379 ± 4293 km from Kerguelen. Females of both colonies travelled even longer distances, 7792 ± 2056 km from the Falklands and 12,257 ± 1422 km from Kerguelen (Table 3) and the exoduses of females were also longer in duration. Likewise, Thin-billed prions from Kerguelen reached longer maximum distances from the colony (males: 2449 ± 668 km, females: 2660 ± 752 km) than Thin-billed prions from the Falklands (males: 1403 ± 763 km, females: 1460 ± 835 km). The mean daily travel speeds were very similar between sexes, but Kerguelen birds travelled faster (Falklands: 285 ± 61 km/day vs. Kerguelen: 405 ± 117 km/day, Table 3).

**Fig. 4:**
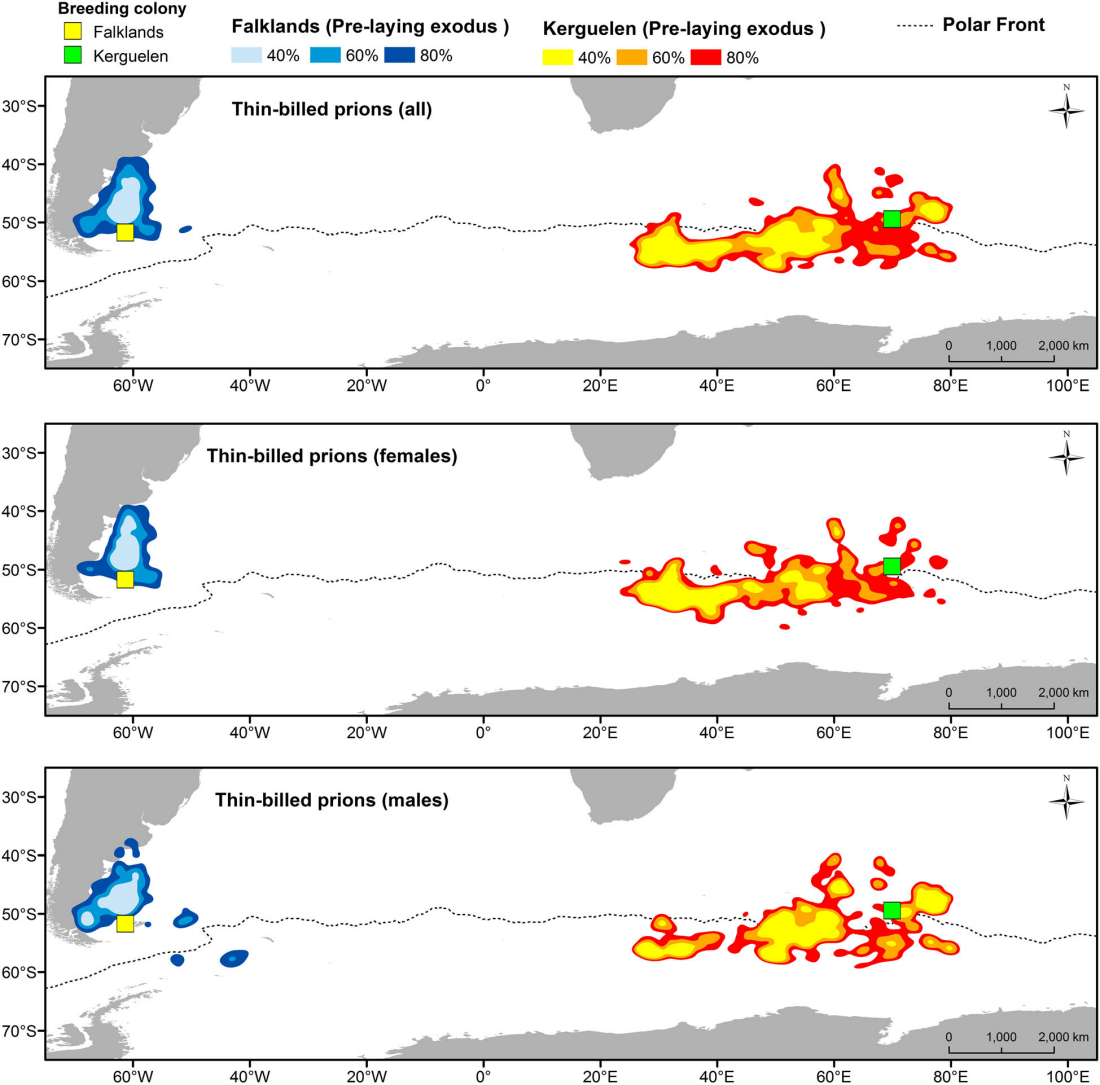
Kernel density contours showing the areas used during the pre-laying exoduses of male and female Thin-billed prions *Pachyptila belcheri* tracked using geolocators from the Falklands (marked light to dark blue) and Kerguelen (marked orange to red). The dotted line represents the oceanographic location of the Polar Front

The distribution during the pre-laying exodus did not differ between the sexes (Fig. 4). Thin-billed prions from the Falklands foraged in temperate waters over the Patagonian Shelf, while Kerguelen birds mainly used open-ocean waters south of the Polar Front (Fig. 4).

### Incubation

The behaviour after return from the pre-laying exodus was consistent between the colonies (Table 4). Females departed earlier on the first incubation trip, after spending 3.2 ± 2.3 days on average in the burrow. Incubation trips by females started on 20 November (± 2 days) on average. Males covered the first incubation shift, thus spending longer than females in the burrow (9.8 ± 2.4 days; Table 4). Males then departed on average on 26 November (± 3 days) for their first incubation trip to sea.

**Table 4:** Effects of colony and sex on timing and duration of the first incubation trip of Thin-billed prions *Pachyptila belcheri* from the Falkland/Malvinas Islands and Kerguelen

Dependent	Independents	F	*P*	Effect size
Period in the burrow after pre-laying exodus	Colony	3.28	0.079	0.086
	**Sex**	**60.79**	**< 0.001**	**0.635**
Departure date	Colony	1.16	0.288	0.032
	**Sex**	**10.54**	**0.003**	**0.231**
Trip duration (days)	**Colony**	**14.7**	**< 0.001**	**0.296**
	Sex	0.58	0.452	0.016
Cumulative travel distance (km)	**Colony**	**32.12**	**< 0.001**	**0.479**
	Sex	0.07	0.794	0.002
Maximum distance from colony (km)	**Colony**	**9.35**	**0.005**	**0.238**
	Sex	0.03	0.869	0.001
Travel speed (km/day)	**Colony**	**12.23**	**0.001**	**0.259**
	Sex	1.9	0.178	0.051

Significant *p*-values are marked bold. As a measure of effect sizes we report partial Eta-Square values (η^2^). None of the interactions were significant (*N* = 50 birds)

The first foraging trips during incubation of males and females were similar in duration, total distance covered and travel speed, but differed between the colonies (Table 4). Incubation trips of Thin-billed prions from Kerguelen were longer in both duration (Table 4, Falklands: 7.3 ± 1.7 days, Kerguelen: 10.3 ± 1.3 days), and total distance covered (Table 4, Falklands: 2737 ± 833 km, Kerguelen: 4850 ± 946 km). Likewise, Thin-billed prions from Kerguelen reached longer maximum distances from the colony during incubation trips (1462 ± 347 km) than Thin-billed prions from the Falklands (883 ± 399 km). Daily travel speeds of males and females were also faster in prions from Kerguelen (Table 4, Falklands: 375 ± 82 km/day, Kerguelen: 495 ± 120 km/day).

**Fig. 5:**
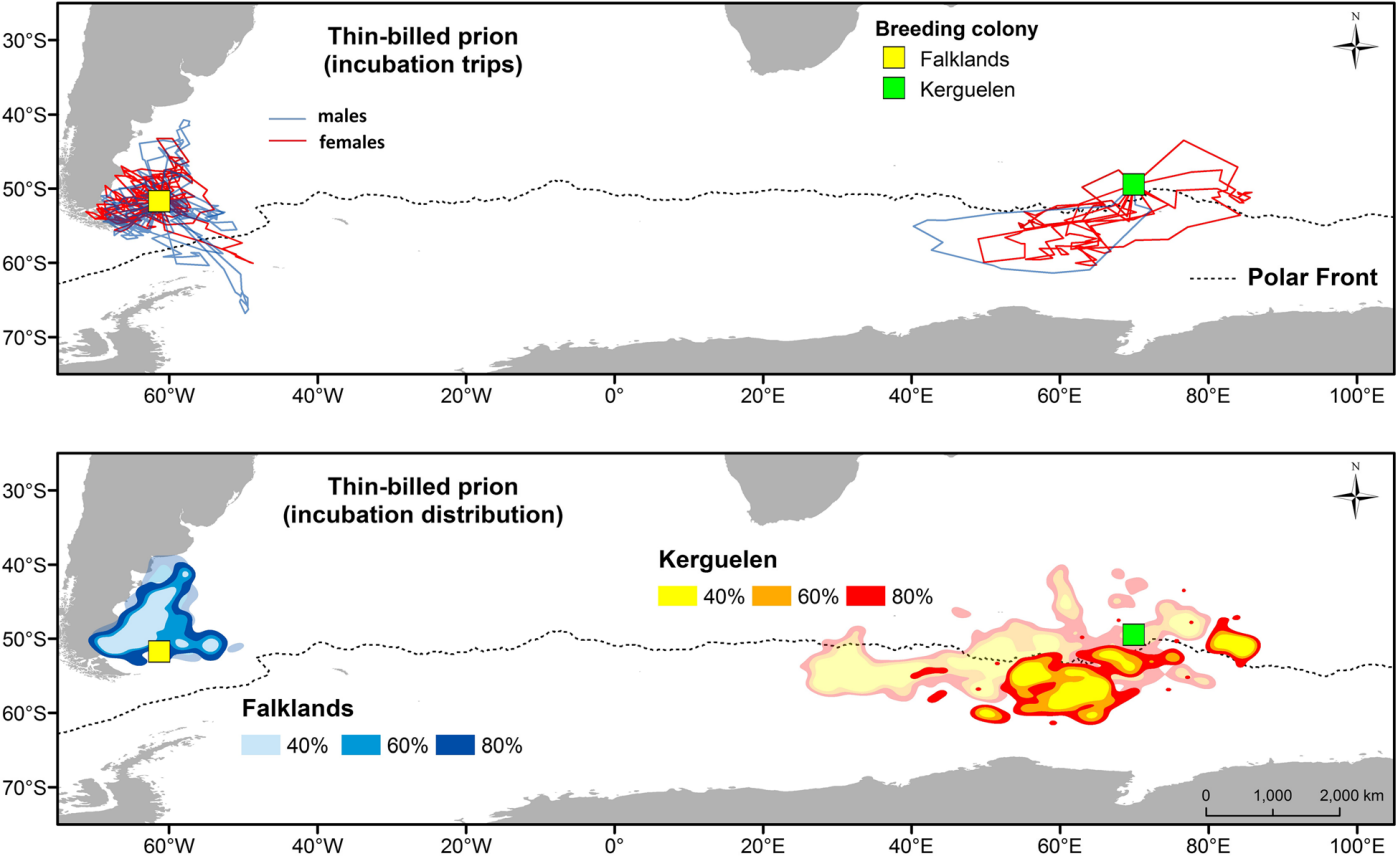
Incubation trips of male and female Thin-billed prions *Pachyptila belcheri* tracked using geolocators. The upper panel shows incubation trips, for females (in red) and males (in blue). The lower panel shows kernel distributions, of birds from the Falklands (marked light to dark blue) and Kerguelen (marked orange to red). For comparison, the kernel distribution during the pre-laying exodus is shown in transparent colors

Thin-billed prions from the Falklands mostly foraged over the Patagonian Shelf during incubation (Fig. 5). Very few individuals crossed the Drake Passage to forage in Antarctic waters south of the Polar Front (three males and one female, Fig. 5). In contrast, all Thin-billed prions from Kerguelen foraged in Antarctic waters south of the Polar Front for most or all of the time (Fig. 5).

### Stable isotope analyses

**Fig. 6:**
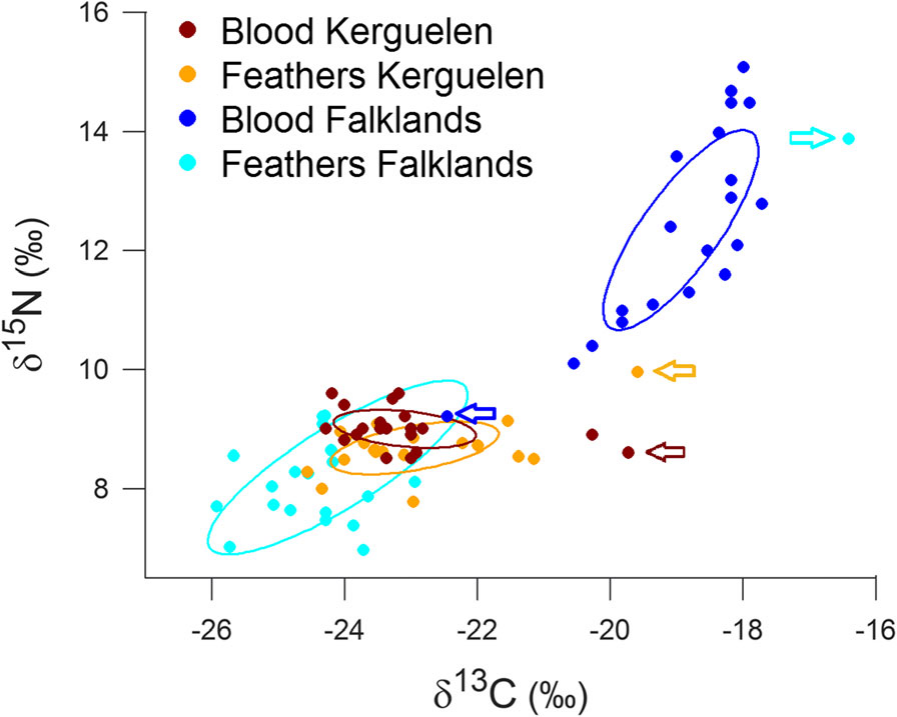
Blood and feather δ^13^C and δ^15^N values of Thin-billed prion *Pachyptila belcheri* from the Falklands (in cyan and blue) and Kerguelen (in orange and red). Feathers are grown during the moult period while blood cells represent the early breeding period. Outliers in δ^13^C values are marked with arrows

**Fig. 7:**
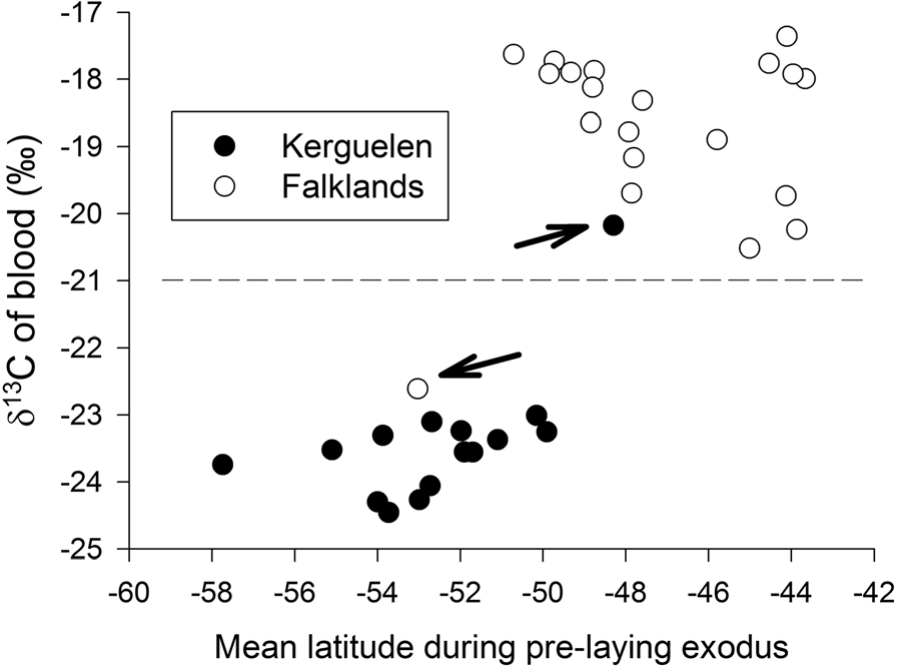
Blood δ^13^C values of Thin-billed prion *Pachyptila belcheri* in relation to the mean latitude registered during the pre-laying exodus trip with geolocators. Outliers in distribution and δ^13^C values are marked with arrows. One additional outlier in blood δ^13^C value marked in Fig. 5 did not have geolocation data during the pre-laying exodus trip

Tissue δ^13^C values reflected a distribution in Antarctic waters for three of four groups: birds from Kerguelen during moult (feathers) and early breeding (blood), and birds from the Falklands during moult (Fig. 6). The only exception were birds from the Falklands, which had blood (i.e. early breeding) δ^13^C values > − 21 ‰. All four groups contained some δ^13^C outliers (Fig. 6, marked with arrows), indicating that these individuals differed in their distribution. The blood values indicated that two birds from Kerguelen spent the early breeding season in subantarctic waters (north of the Polar Front), while one bird from the Falklands went to oceanic waters (Fig. 6). This was also apparent when plotting δ ^13^C values in relation to the latitudinal distribution during the pre-laying exodus (Fig. 7). There were no statistical difference in blood δ^13^C or δ^15^N values between the sexes, either for Kerguelen or the Falklands (all *P* > 0.05).

Feather δ^13^C values indicated that one bird from each colony moulted north of the Polar Front (Figs. 6, 7).

The size of the isotopic niche differed between the two colonies (Fig. 6, Table 5). Kerguelen birds had considerably narrower niches than those from the Falklands, both during moult and the pre-breeding period (Fig. 6). Blood isotopic values from Falkland prions showed a continuous set of values up to remarkably high δ^13^C (> − 18 ‰) and δ^15^N (> 14‰) values (Fig. 6).

**Table 5:** Isotopic niche metrics of Thin-billed prions *Pachyptila belcheri* from the Falkland Islands and Kerguelen, calculated with the SIAR package in R (R Development Core Team 2014)

Symbol	Explanation	Falklands (blood)	Kerguelen (blood)	Falklands (feathers)	Kerguelen (feathers)
LOC	Location of centroid				
	δ^13^C	−18.74	−23.30	−24.41	−23.14
	δ^15^N	12.26	8.90	8.25	8.58
SEA	Area of the standard ellipse (isotope niche width)	4.06	1.11	5.36	1.64
SEAc	as above, corrected for sample size	4.28	1.17	5.66	1.73
NR	trophic length (range in δ^15^N)	5.90	1.10	6.95	2.19
CR^a^	diversity of basal resources (range in δ^13^C)	5.20	5.00	10.45	5.46
CD	niche width 2 (Mean distance to centroid)	1.79	0.81	1.50	1.17
MNND	mean Nearest Neighbour Distance	0.53	0.18	0.80	0.38

^a^including the exceptions (birds with a temperate distribution) marked in Fig. 5

Feathers are grown during the moult period while blood cells represent the early breeding period. The location of the centroid (LOC) indicates where the niche is centered in isotope space. A Bayesian approach based on multivariate ellipse metrics was used to calculate the standard ellipse area SEA, which represents the core isotope niche width [35]. To describe the spread of the data points, parameters proposed by [34] were calculated. As proxies of intrapopulation trophic diversity, the mean distance to centroid (CD) and the mean nearest-neighbour distance (NND) are given. Information on the trophic length of the community is given as the δ15N range (NR), and an estimate of the diversity of basal resources is provided by the δ13C range (CR)

## Discussion

Phenological dynamics such as the timing of migration and breeding determine if there is a match in the resource supply and demand in seasonally breeding birds (e.g [36]). Intraspecific comparisons may be particularly powerful in demonstrating phenotypic plasticity in the timing of migration and breeding. We here found clear differences in the colony attendance patterns and pre-laying movements of Thin-billed prions of the Falklands and Kerguelen, which exhibit differences in their timing and direction of migration. Kerguelen birds spent much less time attending the burrow before their pre-laying exodus, but the onset of incubation was synchronous in both populations. During the pre-laying exodus and incubation, Thin-billed prions from the Falklands spread over the Patagonian Shelf, while prions from Kerguelen aimed for oceanic waters. Although prions from Kerguelen moved much further, their isotopic niches were considerably narrower, suggesting a stronger reliance on polar waters.

### Timing

An appropriate timing of the breeding season within annual cycles is critical to both reproductive success and survival of animals [37]. We used geolocators to compare pre-laying attendance patterns and distributions of male and female Thin-billed prions from the two largest colonies of this species, situated in two oceans. We found clear differences between the Falklands and Kerguelen in the timing of the return to the colony, and the behaviour in the pre-laying phase. This was in strong contrast to the consistent behaviour of Thin-billed prions from the Falklands among years (Fig. 2, [13]). However, the different duration of activities in the sequence of events prior to egg-laying in both colonies finally resulted in a similar onset of incubation in both colonies. Birds from Kerguelen returned later, but then spent little time attending the colony. This suggests a strong selection on arrival date in the Kerguelen population, as birds have only a narrow time window to reunite with their partner and mate, before departing on the long pre-laying exodus.

### Distribution

The foraging distributions and ecological niches during the pre-laying exodus and first incubation trips of tracked birds from the Falkland Islands and Kerguelen differed substantially. Thin-billed prions from the Falklands foraged primarily over neritic waters of the Patagonian shelf, while Kerguelen birds favored oceanic waters of the Antarctic Zone, south of the Polar Front (Figs. 4 and 5). In accordance with the GLS distribution data, blood δ^13^C values indicated that most Kerguelen birds used Antarctic waters to forage during the beginning of the breeding cycle (δ^13^C < − 23‰, Fig. 5; [25]). There they fed primarily on low trophic level prey, most likely crustaceans, as indicated by their relatively low blood δ^15^N values (< 9 ‰; [16]). When compared to Falkland birds, Kerguelen prions had very narrow isotopic niches (Fig. 7, Table 5), indicating much stronger specialization with respect to the water mass used for foraging and the prey type consumed. The inter-breeding (feather) and early breeding (blood) isotopic niches of thin-billed prions from Kerguelen overlapped widely, indicating consistency across time. In contrast, Falkland birds showed wider isotopic niches in both dimensions (δ^13^C and δ^15^N), which did not overlap between feathers and blood, being thus in agreement with birds molting in Antarctic waters [30] and moving afterwards to the Patagonian shelf ([13], this study).

Interestingly, the large range and continuum of blood δ^13^C and δ^15^N values showed that, in early breeding, thin-billed prions from the Falklands foraged in different temperate marine ecosystems marked with different baseline values reflecting the well-known inshore/offshore isotopic gradient from oceanic to truly neritic waters [38]. In some species, such population-related habitat specialization is associated with breeding (or mating) asynchrony, and may promote evolutionary divergence between populations via local adaptation. For example, the two populations of Cook’s petrels *Pterodroma cookii* in New Zealand exhibit habitat specialization during the non-breeding season, associated with one month of asynchrony in migration schedules and breeding timetables, leading to restricted gene flow and genetically distinct populations [39]. In the case of the Thin-billed prions, however, the present study shows breeding synchrony, and a population genetic analysis has shown that gene flow between the two populations is large enough to maintain a genetically unstructured species [40].

A few individuals (< 10%) of both populations also showed some contrasting distribution (marked with arrows in Fig. 5) to the remaining population. Previous analyses have also found that small number (7–14%) of Thin-billed prions from the Falkland Islands differed from the remaining population in the habitat used during moult [27, 41], and analyses of individuals over several years have shown that this was caused by phenotypic plasticity [26]. As Falkland Islands birds have wide isotopic niches and change between habitats during the breeding season, such plasticity is not too surprising. In particular, it is caused by birds that do not migrate (i.e. stay the whole inter-breeding period over the Patagonian shelf). Analyses of museum feathers further showed that this strategy was more common in the past, when Patagonian shelf waters were possibly more favourable foraging grounds in winter [41]. In the same way, feathers from museum specimens showed that historically Thin-billed prions from Kerguelen moulted in subantarctic waters and recently shifted their moulting grounds further south [16]. Furthermore, inter-annual variability in the timing of breeding according to the environmental conditions has been observed in Thin-billed prions [42]. Overall, time- and spatial-related comparisons of Falkland and Kerguelen birds highlight the foraging plasticity of the Thin-billed prion in relation to the local breeding environment and environmental changes that affect it.

## Conclusions

The different phenology and distribution patterns in the two populations suggest local adaptation to different environmental conditions. The differences found in the present study suggest that seabirds using different environments (e.g. shelf vs. open ocean) may offer a good study system to understand mechanisms of the timing of reproduction under variable conditions.

## Additional file

Additional file 1: **Figure S1.** Independent pre-laying exodus trips of male and female Thin-billed prions *Pachyptila belcheri* tracked using geolocators. The upper panel shows paired pre-laying exodus trips of three Thin-billed prion pairs, from New Island (Falkland Islands) for females (in red) and males (in blue). The lower panel shows two Kerguelen pairs. (PDF 184 kb)
