# Supplementary material for: Behavioural plasticity in the early breeding season of pelagic seabirds - a case study of thin-billed prions from two oceans
Source: Mov Ecol. 2019 Jan 22;7:1. doi: 10.1186/s40462-019-0147-7 (PMC6341530; doi:10.1186/s40462-019-0147-7)
Supplement: Supplementary file 1 — Figure S1. Independent pre-laying exodus trips of male and female Thin-billed prions Pachyptila belcheri tracked using geolocators. The upper panel shows paired pre-laying exodus trips of three Thin-billed prion pairs, from New Island (Falkland Islands) for females (in red) and males (in blue). The lower panel shows two Kerguelen pairs. (PDF 184 kb) [file 40462_2019_147_MOESM1_ESM.pdf]

Behavioural plasticity in the early breeding season of pelagic seabirds - a case study of Thin-billed prions from two oceans

Petra Quillfeldt, Henri Weimerskirch, Juan F. Masello, Karine Delord, Rona A.R. McGill, Robert W. Furness & Yves Cherel

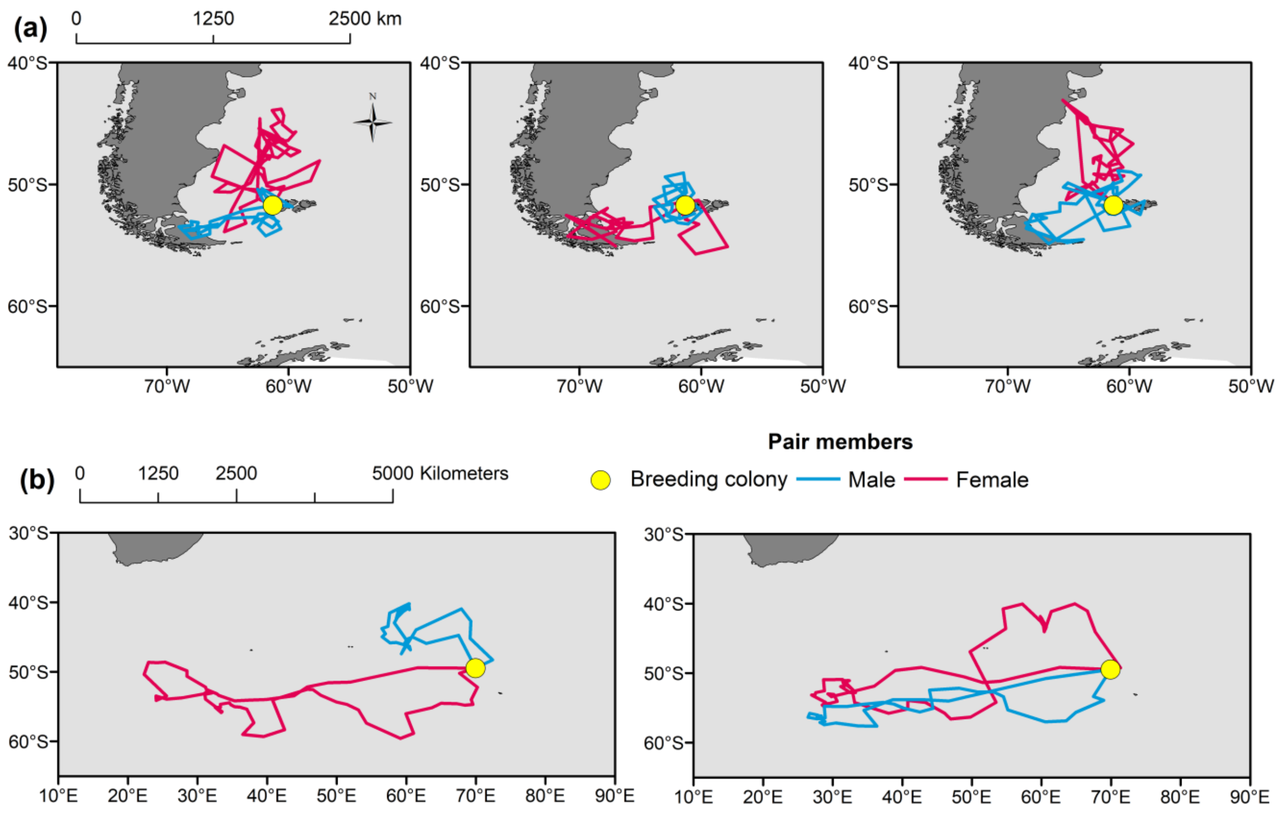

Fig. S1. Independent pre-laying exodus trips of male and female Thin-billed prions *Pachyptila belcheri* tracked using geolocators. The upper panel shows paired pre-laying exodus trips of three Thin-billed prion pairs, from New Island (Falklands Islands) for females (in red) and males (in blue). The lower panel shows two Kerguelen pairs.
